# Promising new vaccine candidates against *Campylobacter* in broilers

**DOI:** 10.1371/journal.pone.0188472

**Published:** 2017-11-27

**Authors:** Marine Meunier, Muriel Guyard-Nicodème, Estelle Vigouroux, Typhaine Poezevara, Véronique Beven, S. Quesne, Lionel Bigault, Michel Amelot, Daniel Dory, Marianne Chemaly

**Affiliations:** 1 HQPAP–Unit of Hygiene and Quality of Poultry and Pork Products, French Agency for Food, Environmental and Occupational Health & Safety (ANSES), Ploufragan, France; 2 GVB–Viral Genetics and Biosafety Unit, French Agency for Food, Environmental and Occupational Health & Safety (ANSES), Ploufragan, France; 3 SELEAC—Avian Breeding and Experiment Department, French Agency for Food, Environmental and Occupational Health & Safety (ANSES), Ploufragan, France; University of Campinas, BRAZIL

## Abstract

*Campylobacter* is the leading cause of human bacterial gastroenteritis in the European Union. Birds represent the main reservoir of the bacteria, and human campylobacteriosis mainly occurs after consuming and/or handling poultry meat. Reducing avian intestinal *Campylobacter* loads should impact the incidence of human diseases. At the primary production level, several measures have been identified to reach this goal, including vaccination of poultry. Despite many studies, however, no efficient vaccine is currently available. We have recently identified new vaccine candidates using the reverse vaccinology strategy. This study assessed the *in vivo* immune and protective potential of six newly-identified vaccine antigens. Among the candidates tested on Ross broiler chickens, four (YP_001000437.1, YP_001000562.1, YP_999817.1, and YP_999838.1) significantly reduced cecal *Campylobacter* loads by between 2 and 4.2 log_10_ CFU/g, with the concomitant development of a specific humoral immune response. In a second trial, cecal load reductions results were not statistically confirmed despite the induction of a strong immune response. These vaccine candidates need to be further investigated since they present promising features.

## Introduction

Since 2005 in Europe, *Campylobacter* is the most reported gastrointestinal bacterial pathogen in humans. More than 229,000 cases were reported in 2015 [1], though it was estimated that *Campylobacter* affects approximately nine million people each year [2]. Campylobacteriosis is generally a self-limited disease causing abdominal pain, diarrhea and fever, but it can also lead to extra-intestinal manifestations such as bacteremia or neurological disorders [3].

Human infections are mainly associated with handling and/or consuming raw or undercooked poultry meat [1]. Birds are described as the main reservoir of the pathogen, carrying it commensally within their intestines. The chicken reservoir as a whole could account for 50–80% of cases [2, 4] and broiler meat could be responsible for 20–30% of human campylobacteriosis. The prevalence of *Campylobacter* in primary poultry production is very high in Europe, with up to 70% of broiler batches infected and about 75% of broiler carcasses being contaminated at the slaughterhouse [5]. *Campylobacter jejuni* and *Campylobacter coli* are mainly responsible for human diseases causing approximately 90% and 10% of cases respectively. It was estimated that a reduction in cecal poultry colonization of about 2 to 3 log_10_ CFU/g of cecal contents could reduce the risk of human campylobacteriosis by 76 to 100% [6, 7]. At the primary production level, several measures have been identified and studied in order to significantly reduce *Campylobacter* loads in live birds, including hygiene and biosecurity measures or nutritional and immune strategies [8]. However, despite promising results, there is currently no measure relevant to large-scale applications that are able to decrease *Campylobacter* avian gut colonization and thus impact the incidence of human disease.

One of the promising ways to decrease intestinal *Campylobacter* loads is to vaccinate poultry, a measure that has been widely studied over the past few decades, providing descriptions of many different strategies. Globally, the first experiments used whole-cell vaccines [9–13], whereas more recent studies have been performed with subunit vaccines [14–19] or microorganism-vectored vaccines [20–24]. Although several vaccine candidates offered a decrease in *Campylobacter* colonization levels [19, 23], there is as yet no vaccine available on the market to reduce the intestinal colonization of broilers by *Campylobacter*. There is still a need to identify and evaluate new vaccine antigens.

We recently identified new vaccine candidates against *Campylobacter* using the reverse vaccinology strategy [25]. After analyzing the *C*. *jejuni* genome (strain 81–176) encoding more than 1,700 proteins, we selected 14 antigens based on their subcellular localization, global immunogenicity, B-cell epitopes density and their sequence conservation among *C*. *jejuni* and *C*. *coli* strains. The identified proteins were either flagellar-associated proteins, proteins with other functions or hypothetical proteins. (26)The objective of this study was to assess on live broilers the immune and protective powers of six out of 14 antigens (putative OMP YP_001000437.1, flagellin protein family YP_001000562.1, flagellar hook-associated protein YP_0010001115.1, flagellar hook protein YP_999769.1, hypothetical proteins YP_999817.1 and YP_999838 .1) identified *in silico* [25] and using a DNA prime / protein boost vaccine regimen.

## Materials and methods

### *Campylobacter* strains and growth

*Campylobacter jejuni* strain 81–176 was used for vaccine production and extraction of the total proteins used in ELISAs (detection of humoral immune responses in chicken sera). Strain C97Anses640, isolated from a poultry product and belonging to the ST-45 complex, was used for the oral *in vivo* challenge. Strains were cultured under microaerophilic conditions (85% N_2_, 10% CO_2_ and 5% O_2_) at 41.5°C on *Campylobacter* charcoal differential agar media (mCCDA) for 48 h or in Brucella broth for 24 h.

### Recombinant plasmid constructs

From *C*. *jejuni* 81–176 genomic DNA, genes encoding YP_001000437.1, YP_001000562.1, YP_001001115.1, YP_999769.1, YP_999817.1, and YP_999838.1 from the Vaxign database (named YP437, YP562, YP1115, YP9769, YP9817, and YP9838 hereinafter) were amplified by Polymerase Chain Reaction (PCR) (primers listed in Table 1), using the Vent^®^ DNA polymerase (NEB) or the Platinium^®^ *Taq* polymerase high fidelity (Invitrogen). A thermal cycler (Eppendorf, Montesson, France) was used to implement the following temperature cycles: 95°C for 5 min, followed by 30 cycles of 30 s at 95°C, 30 s at adequate Tm (Table 1), 1 min/kb at 72°C for the Vent polymerase or 68°C for the Taq polymerase, and a final extension step of 5 min at 68 or 72°C.

**Table 1:** Primers used for the PCR amplification of *Campylobacter* genes encoding new vaccine candidates. Restriction sites inserted in each primer (BamHI: GGATCC, NotI: GCGGCCGC, NcoI: CCATGG, SacI: GAGCTC) are displayed in bold type.

Primer	Sequence	Cycling conditions	Polymerase
*pcDNA3 cloning*			
YP437_F1	5'-GCCGCC**GGATCC**ATGAGAAAAATTTTAGTTGTTTTAG-3'	95°, 30 s/ 52°C, 30 s/ 72°C, 2 min	Vent
YP437_R1	5'-CCGCCG**GCGGCCGC**TTAATATTCTTTATTTTCATACC-3'		
YP562_F1	5'-GCCGCC**GGATCC**ATGAGAATTACAAATAAACTTAAC-3'	95°, 30 s/ 55°C, 30 s/ 72°C, 2 min 30 s	Vent
YP562_R1	5'-CCGCCG**GCGGCCGC**TTACATATAATTTAATAAGCTAAG-3'		
YP1115_F1	5'-GCCGCC**GGATCC**ATGGGTATTTTTGGAACATTATAC-3'	95°, 30 s/ 50°C, 30 s/ 72°C, 2 min	Vent
YP1115_R1	5'-CGGCGG**GCGGCCGC**TTAAGATTTAAGCCCTAGTAAGG-3'		
YP9769_F1	5'-GCCGCC**GGATCC**ATGATGAATTCATTTTATAATGGG-3'	95°, 30 s/ 50°C, 30 s/ 72°C, 2 min	Vent
YP9769_R1	5'-CGGCGG**GCGGCCGC**TTATCTTTTCATATTAATGGC-3'		
YP9817_F1	5'-GCCGCC**GGATCC**ATGAAAATAATTAAAATTCTTTTTTTAGG-3'	95°, 30 s/ 57°C, 30 s/ 68°C, 1 min 30 s	Taq HF
YP9817_R1	5'-CCGCCG**GCGGCCGC**TTAAAAGCCTAGATTTACTCCGCC-3'		
YP9838_F1	5'-GCCGCC**GGATCC**ATGAAAAAAATATTCACAGTAGC-3'	95°, 30 s/ 50°C, 30 s/ 68°C, 1 min 30 s	Taq HF
YP9838_R1	5'-CCGCCG**GCGGCCGC**TTATTTTCTATTAGGTGAAGC-3'		
*pQE-trisystem cloning*			
YP437_F2	5'-GCCGCC**CCATGG**GCAGAAAAATTTTAGTTGTTTTAG-3'	95°, 30 s/ 52°C, 30 s/ 72°C, 2 min	Vent
YP437_R2	5'-CCGCCG**GCGGCCGC**ATATTCTTTATTTTCATACC-3'		
YP562_F2	5'-GCCGCC**CCATGG**GCAGAATTACAAATAAACTTAAC-3'	95°, 30 s/ 53°C, 30 s/ 68°C, 2 min 30 s	Taq HF
YP562_R2	5'-CCGCCG**GCGGCCGC**CATATAATTTAATAAGCTAAG-3'		
YP1115_F2	5'-GCCGCC**GAGCTC**GGTATTTTTGGAACATTATAC-3'	95°, 30 s/ 60°C, 30 s/ 72°C, 2 min	Vent
YP1115_R2	5'-CGGCGG**GCGGCCGC**AGATTTAAGCCCTAGTAAGG-3'		
YP9769_F2	5'-CGGCCG**CCATGG**CCATGAATTCATTTTATAATGGG-3'	95°, 30 s/ 50°C, 30 s/ 72°C, 2 min	Vent
YP9769_R2	5'-CCGCCG**GCGGCCGC**TCTTTTCATATTAATGGC-3'		
YP9817_F2	5'-GCCGCC**CCATGG**GCAAAATAATTAAAATTCTTTTTTTAGG-3'	95°, 30 s/ 57°C, 30 s/ 68°C, 1 min 30 s	Taq HF
YP9817_R2	5'-CCGCCG**GCGGCCGC**AAAGCCTAGATTTACTCCGCC-3'		
YP9838_F2	5'-GCCGCC**CCATGG**GCAAAAAAATATTCACAGTAGC-3'	95°, 30 s/ 51°C, 30 s/ 72°C, 1 min 30 s	Vent
YP9838_R2	5'-CCGCCG**GCGGCCGC**TTTTCTATTAGGTGAAGCTTTTG-3'		

Purified amplicons were digested by appropriate restriction endonucleases **(Table 1)** for pcDNA3 or pQE-Trisystem (pQE-TS, Qiagen) cloning.

*Escherichia coli (E*. *coli)* Top10 (Invitrogen) or XL-1 Blue were respectively transformed with pcDNA3-YP to produce the DNA vaccine or with pQE-TS-YP to produce the recombinant protein. After individual culturing of clones on agar plates and then in LB broth (Sigma, Lyon, France) with adequate antibiotics, recombinant plasmids were extracted from bacterial clones and checked for correct sequence by enzymatic digestion and sequencing (3130 Genetic Analyzer, ThermoFischer Scientific). Validated clones were used for vaccine production.

### Production of DNA vaccines

DNA vaccines pcDNA3-YP and pcDNA3 were produced using NucleoBond Endotoxin Free extraction kits (Macherey-Nagel) according to the manufacturer’s recommendations. Plasmid concentrations were assessed by measuring the optical density (OD) at 260 nm. Plasmids were stored at -20°C until vaccination.

### Production of the protein vaccine

The QIAexpressionist protocol (Qiagen) was used to produce the recombinant proteins according to the manufacturer's recommendations. The proteins produced in pQE-TS harbored a Histidine Tag on the C-terminal end.

Globally, *E*. *coli* XL-1 Blue, transformed by pQE-TS-YP, were cultured on LB broth supplemented with ampicillin (50 μg/mL) and tetracycline (25 μg/mL) until they reached an OD of 0.6 at 600 nm. Protein production was then induced by adding 1 mM of isopropyl-β-D-thiogalactoside (IPTG, Sigma). The culture was extended for 5 h and the cells were harvested by centrifugation for 20 min at 4 000 *g*. For large-scale protein production, transformed *E*. *coli* XL-1 Blue were cultured in 1 L of LB broth. Recombinant proteins were purified differently depending whether they were recovered from the soluble protein fraction (YP562, YP9769, YP9817 and YP9838) or the insoluble one (YP437 and YP1115).

For recombinant soluble proteins, the cell pellet was resuspended in a lysis buffer (50 mM NaH2PO4, 300 mM NaCl and 10 mM imidazole, pH 8). Lysozyme (1 mg/mL) was added and the suspension was incubated on ice for 30 min before sonication using a Bioruptor UCD300 (Diagenode) and performing 10 cycles (30 sec ON/30 sec OFF). Lysate was centrifuged for 30 min at 10 000 *g* and 4°C. The supernatant containing the recombinant protein was removed. Affinity chromatography was used for protein purification by FPLC (Akta FPLC, Amersham biosciences) with HisTrap HP columns (GEHealthCare Life Sciences). Purified proteins were dialyzed against PBS and stored at -80°C.

For the recombinant insoluble proteins, the cell pellet was resuspended in an 8 M urea buffer containing 100 mM NaH_2_PO_4_ and 10 mM Tris-Cl, pH 8. After stirring at room temperature until total lysis, the suspension was centrifuged at 10 000 *g* for 30 min and the supernatant harvested. Thereafter, the protein solution was progressively dialyzed using decreasing urea concentrations from an 8 M urea buffer to a PBS buffer and stored at -80°C.

### Assessment of avian vaccine efficiency

Vaccine experiments were approved by the ANSES ethical committee (ethical protocol no. 14–065, agreement no. 09/12/14-1B) (S1 Table).

A total of 314 day-of-hatch Ross PM3 broiler chicks (male and female) were purchased from a local hatchery (Perrot hatchery, France). Before their arrival, chicks had been vaccinated against infectious bronchitis. Birds were randomly allocated into groups of 16 or 20 and kept in floor pens with unused wood shavings. With 16 birds per group, the power calculation was estimated to be of 0.8. Both the birds and the environment were confirmed *Campylobacter-*free at the beginning of the experiments.

The chicks were vaccinated by the intramuscular route (IM) on day 5 with 300 μg of pcDNA3-YP, supplemented with 50 μg of unmethylated CpG ODN2007 (TCGTCGTTGTCGTTTTGTCGTT, with a phosphorothioate backbone, Sigma) as an adjuvant and on day 12, with 100 μg of recombinant proteins emulsified in MONTANIDE™ ISA70 VG (SEPPIC). Similarly, in the DNA/protein control group, chicks were injected with pcDNA3 supplemented with CpG ODN2007, then with PBS emulsified in MONTANIDE™. The birds were orally challenged by 10^5^ CFU of *C*. *jejuni* C97Anses640 on day 19. On day 22, *C*. *jejuni* intestinal colonization was assessed in 3 to 4 birds in DNA/protein control groups. Systemic immune response was assessed weekly by blood sampling at the occipital sinus on chickens under 21 days (samples of 200 μL) or the wing vein for the last sample points (samples of 500 μL). On day 42 (± 1 day), all the birds were humanely euthanized (electronarcosis followed by bleeding) and ceca were sampled for *Campylobacter* enumeration.

The first trial evaluated six antigens (YP437, YP562, YP1115, YP9769, YP9817, and YP9838) individually or as a mixed pool (50 μg of each DNA plasmid on day 5 followed by 16.7 μg of each recombinant protein on day 12).

The second trial was designed to retest a sub-selection of the best antigens. Four out of the six original antigens (YP437, YP562, YP9817, and YP9838) were evaluated individually and compared to a DNA/protein control group as described above. A negative control group was injected only with PBS on days 5 and 12 (birds received neither antigens nor adjuvant). In this second experiment, additional groups were set up using the YP9817 antigen to assess DNA or protein vaccination alone. One group was vaccinated with pcDNA3-YP9817 supplemented with CpG on day 5. Another one was vaccinated with MONTANTIDE™ emulsified YP9817 on day 12. Corresponding control groups (DNA control group: birds injected with pcDNA3 + CpG on day 5; Protein control group: birds injected with MONTANTIDE™ emulsified PBS on day 12) were also set up.

### Assessment of serum IgY levels by enzyme-linked immunosorbent assays (ELISA)

The birds’ systemic immune responses against *Campylobacter* were assessed by ELISAs on serum. Maxisorp^®^ 96-well plates (Nunc) were coated overnight with 4 μg/ml of *C*. *jejuni* 81–176 total proteins and successively incubated with serial dilutions from 1:100 to 1:800 of tested sera, goat anti-chicken IgY-HRP (horseradish peroxidase) antibody (diluted 1:5000 or 1:15000, depending on the lot, Abcam), o-Phenylenediamine dihydrochloride (OPD, Sigma) substrate in citrate buffer containing hydrogen peroxide and 1 M H_2_SO_4_. The OD was measured at 490 nm using a spectrophotometer (Infinite 200 PRO NanoQuant, Tecan, Lyon, France).

### Assessment of the protective response against *Campylobacter* by cecal enumeration or qPCR

*Campylobacter* were enumerated in ceca using bacterial culture (trial 1) or qPCR (trial 2). *Campylobacter* enumerations from ceca samples were performed as described by Guyard-Nicodeme et al. (2016). Ceca were collected at necropsy and diluted (1:10) in Tryptone salt broth. After homogenization, cecal content samples were serially diluted in Tryptone salt broth (Biomerieux) and plated on mCCDA medium using easySpiral^®^ (Interscience). *Campylobacter* colonies were enumerated on plates after incubation for 48 h ± 5 h at 41.5°C under microaerophilic conditions (85% N2, 10% CO2 and 5% O2). Counts were converted to log_10_ (CFU/g) in cecal content loads.

For qPCR enumeration, 1 mL of the first dilution from each sample in Tryptone salt broth was centrifuged at 10 000 *g* for 10 min and the pellet was conserved at -20°C for further molecular analysis. Genomic DNA was extracted from the 1 mL frozen pellet from the first dilution of cecal content as described by Saint-Cyr et al. (2016). Briefly, the NucleoMag 96^®^ Tissue kit (Macherey-Nagel) was used with modifications. To improve the cell lysis step, 100 mg of 0.1 mm zirconium/silica beads were added and homogenized using a TissueLyser II (Qiagen). DNA was extracted from the supernatants using the KingFisher Duo magnetic particle processor (Thermo Fisher Scientific). DNA samples were stored at −20°C until molecular analyses.

To examine the *C*. *jejuni* level in cecal contents, quantification by real-time PCR using rpoA primers (rpoA-F: GCACCATAGGATATGCTCCAACT and rpoA-R: CCACGCATGCTATCAAATTCAT [27]) was performed on a BioRad CFX96™Real-Time PCR Detection System in Hard-Shell^®^ 96-Well PCR plates (Bio-Rad). PCR reactions were performed in 25 μL PCR volumes containing SYBR® Green JumpStart™ TaqReadyMix™ (Sigma-Aldrich) 1 X, 0.3 μM of each primer and 10 μL of DNA template (at 10 ng/mL). The temperature cycling parameters were 2 min at 94°C then 40 cycles consisting of 94°C for 15 s followed by 60°C for 1 min. The cycle threshold (Ct) was manually determined for each run by the user. A DNA sample extracted from a fresh pure culture of *C*. *jejuni* was used as the real-time PCR standard (from 5.62 to 5.62 x 10^6^ genome equivalents (GenEq) per reaction). Bacteria were quantified using the standard curve method, and all reactions were performed in duplicate. The qPCR method for enumerating *Campylobacter* had been previously validated and demonstrated a good correlation with the conventional microbiological method [28].

### Statistical analysis

R software [29] available at http://www.r-project.org/ was used for statistical analysis. To compare bacterial loads and antibodies levels between groups, the Kruskal-Wallis test was applied as the data were not normally distributed and the variance was not homogenous (checked by the Shapiro-Wilk normality test and Bartlett’s test respectively). Significant differences (p < 0.05) between vaccinated groups and the control were estimated using the Wilcoxon test.

## Results

### Assessment of the immune response

**Fig 1:**
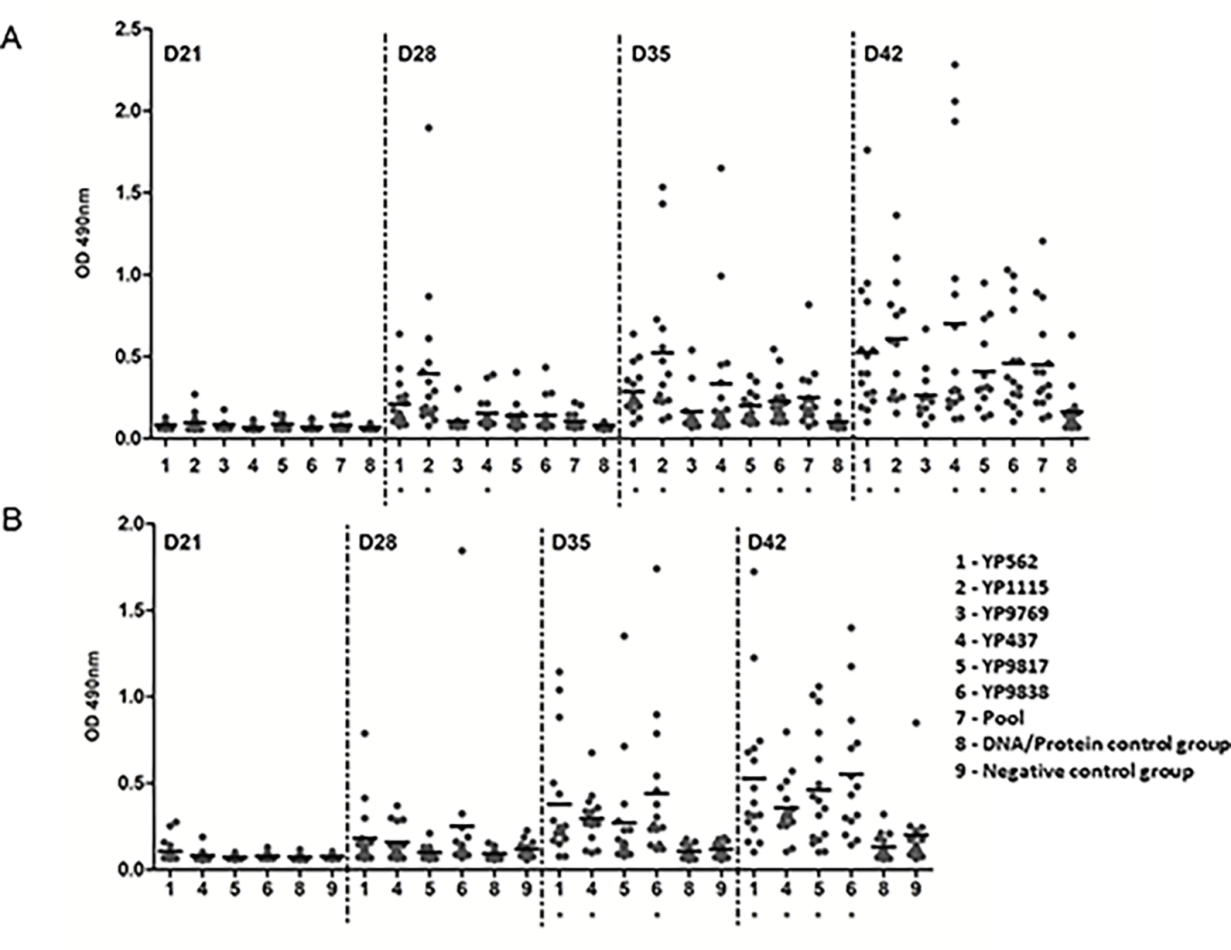
Anti-*Campylobacter* IgY antibodies in the sera of chickens after vaccination and challenge. The chicks were immunized by the IM route, on day 5 with DNA and on day 12 with proteins. They were then orally infected on day 19 by *Campylobacter*. The specific systemic immune response was assessed weekly from blood samples until the end of the experiment on day 42 ± 1 day (data from day 1 to day 14 not shown) by ELISAs which reveal the IgY antibodies (OD 490nm) (A) YP562, YP1115, YP9769, YP437, YP9817, and YP9838 antigens were individually injected in the first experiment along with a combination of antigens (Pool) and compared to a DNA/protein control group (injected with adjuvants only). (B) Four antigens (YP562, YP437, YP9817, and YP9838) were tested again in a second experiment and compared to a DNA/protein control group (injected with adjuvants only). The negative control group was administered with PBS only. Each dot corresponds to the OD of an individual chicken. Bars represent the medians for each group. *: statistically significant differences from the control group at the same time point (p < 0.05).

To assess the effect of the six antigens (YP437, YP562, YP1115, YP9769, YP9817, and YP9838) on chicken immune response, a first *in vivo* trial was carried out. The six antigens were injected individually or in combination into live Ross broiler chicks following the DNA/recombinant protein regimen described above. Thereafter, the birds were orally challenged with *Campylobacter* on day 19. On day 21, 4 chickens in DNA/protein control group were sacrificed in order to measure the *Campylobacter* cecal colonization: the values ranged from 4.4 to 7.5 log_10_ CFU/g. CFU/g. This shows that the *Campylobacter* inoculation was done properly. Specific humoral responses were assessed weekly by ELISAs. As the aim of the present study was to screen the immune potentials of 6 antigens, we decided to use an ELISA prepared with total proteins of *Campylobacter* (therefore compatible with each studied antigen). As shown in Fig 1A, adjuvants alone were not able to induce a specific humoral immune response at any time-point (DNA/protein control group). On the other hand, specific IgY levels increased significantly from day 28, i.e. 16 days after immunization, for three out of the six antigens investigated (YP437, YP562, and YP1115) compared to the DNA/protein control group. On days 35 and 42, six out of seven vaccinated groups showed significantly higher levels of IgY than the DNA/protein control group. The mean OD on day 42 was 0.53, 0.61, 0.70, 0.41, 0.46 and 0.45 for the YP562, YP1115, YP437, YP9817, YP9838, and Pool groups respectively, whereas the DNA/protein control group showed a mean OD of only 0.16. The YP9769 antigen did not allow the development of a significant specific humoral response in serum (mean OD on day 42 of 0.26).

In the second trial, the effect of the four antigens YP437, YP562, YP9817, and YP9838 was evaluated again using the same vaccine regimen (Fig 1B). The colonization control performed on day 21 on 3 chickens in the DNA/protein control group indicated that the *Campylobacter* cecal contents ranged from 7.9 to 8.8 log_10_ CFU/g, showing that the *Campylobacter* inoculation was also done properly. The same trends were observed, confirming the immune potential of these four vaccine candidates. During this second trial, a negative control group (without antigen nor adjuvant) was added and confirm that the observed IgY response in vaccinated groups was due to the antigens and not to the adjuvants since this negative control group showed similar low IgY levels as the DNA/protein control group (without antigen but with adjuvant) for all the sampling time points, indicating that adjuvants alone were not able to induce specific humoral response (Fig 1B).

### Assessment of the protective effect against *Campylobacter* colonization

**Fig 2:**
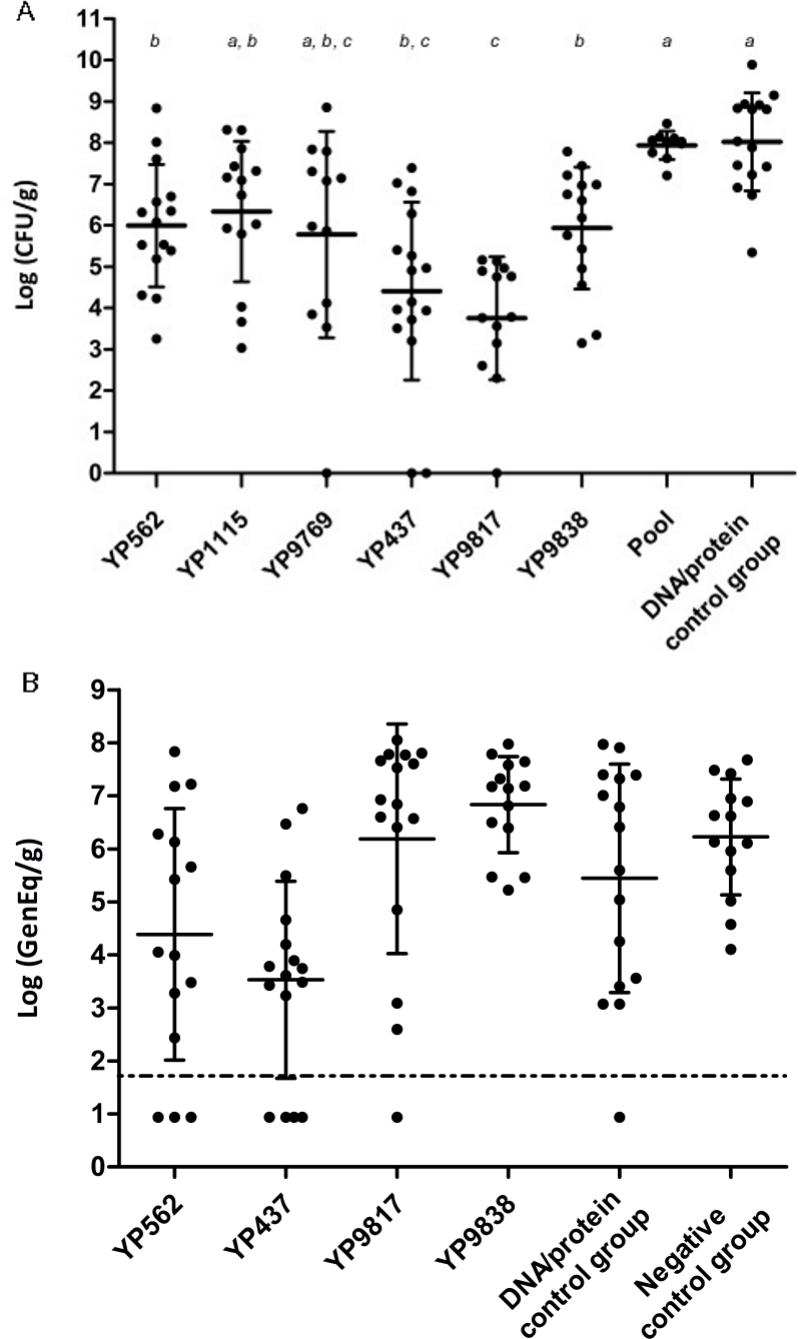
*Campylobacter* loads in chicken ceca after vaccination and challenge. After two vaccinations on days 5 and 12, chickens were then orally infected on day 19. *Campylobacter* counts were evaluated at the end of the experiment from ceca samples. (A) YP562, YP1115, YP9769, YP437, YP9817, and YP9838 antigens were individually injected in the first experiment along with a combination of antigens (Pool) and compared to a DNA/protein control group (injected with adjuvants only). Ceca samples were cultured on mCCDA for *Campylobacter* counts (Log CFU/g). Groups without common letters (a-c) differed significantly (p < 0.05). (B) Four antigens (YP562, YP437, YP9817, and YP9838) were tested again in a second experiment and compared to DNA/protein control group (injected with adjuvant only) and negative control group administered with PBS only. Quantitative PCR on ceca samples was used for *Campylobacter* counts (Log GenEq/g). The detection limit was determined to lie at 1.88 log_10_ GenEq/g. Samples with counts under the threshold were set at 0.94 log_10_ GenEq/g. No significant differences were observed between groups.

Effect of the vaccination strategy on *Campylobacter* colonization was achieved by measuring *Campylobacter* loads at the end of rearing. In the first trial, significant decreases in *Campylobacter* counts were observed in the ceca of four out of six vaccinated groups, as shown in Fig 2A. *Campylobacter* loads were significantly lower for YP562 (5.99 ±1.48 log_10_ CFU/g), YP437 (4.41 ±2.15 log_10_ CFU/g), YP9817 (3.75 ±1.49 log_10_ CFU/g), and YP9838 (5.94 ±1.48 log_10_ CFU/g) antigens than the DNA/protein control group (8.02 ±1.19 log_10_ CFU/g). The largest decrease was 4.2 log_10_ CFU/g for the YP9817 antigen. Despite no significant difference compared to the DNA/protein control group, YP1115 and YP9769 groups tended to be less colonized (p = 0.09 and 0.11 respectively). Contrary to the individually tested antigens, the combination of antigens (Pool, Fig 2A) did not lead to a decrease in the *Campylobacter* count despite inducing a specific humoral response (Fig 1A).

To confirm these promising results a second trial was carried out. During this second trial, the same methodology was applied for *Campylobacter* enumeration (dilution method and plating on mCCDA). Unfortunatly, the growth of other commensal flora on mCCDA plates prevented the enumeration of characteristic *Campylobacter* colonies. Therefore, samples were analyzed using an alternative method known to generate results correlated with *Campylobacter* counts enumeration: a qPCR method [28]. High inter-individual variability was observed among the groups. Even though the protective effect of the YP437 and YP562 antigens seemed to be observed (3.53 ±1.86 and 4.39 ±2.37 log_10_ GenEq/g, respectively, vs 5.45 ±2.16 log_10_ GenEq/g for the DNA/protein control group) (Fig 2B), *Campylobacter* loads were not significantly reduced. On the other hand, and on the contrary to what was observed during the first trial, a non-significant increase in *Campylobacter* loads was observed (6.19 ±2.16 and 6.83 ±0.91 log_10_ GenEq/g respectively) in the groups vaccinated by the YP9817 and YP9838 antigens.

### Importance of the DNA prime / protein boost

**Fig 3:**
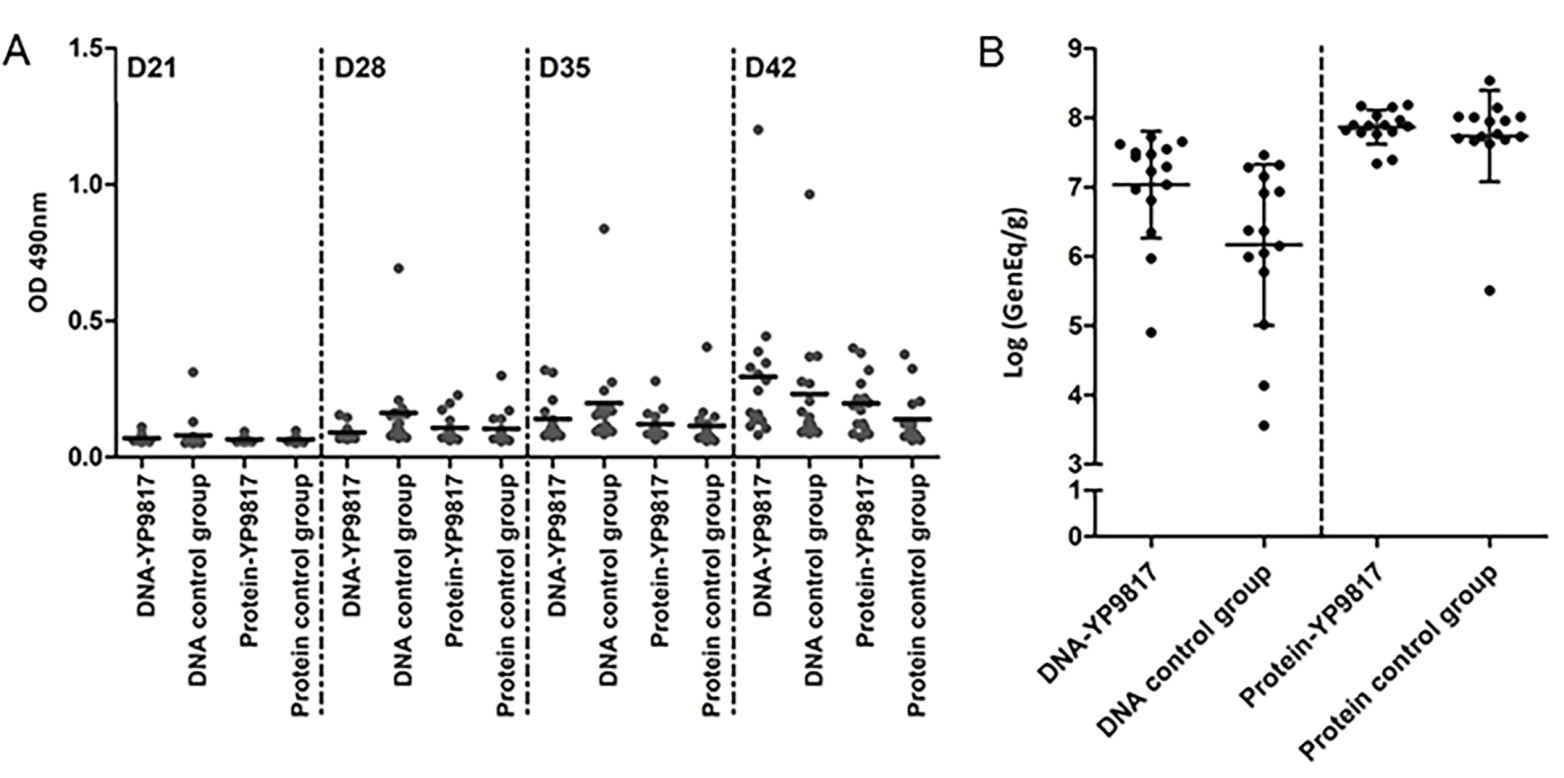
Anti-*Campylobacter* IgY antibodies in sera and *Campylobacter* loads in chicken ceca after DNA vaccination alone or protein vaccination alone and challenged using the YP9817 antigen. Birds were vaccinated with the DNA vaccine alone on day 5 or with proteins alone on day 12 and then infected on day 19. (A) The IgY levels in blood samples were assessed by ELISAs each week until the end of the experiment on day 42 ± 1 day (data from day 1 to day 14 not shown). Each dot corresponds to the OD of an individual chicken. Bars represent the medians for each group. (B) *Campylobacter* counts were evaluated at the end of the experiment from ceca samples by qPCR (Log GenEq/g). The detection limit was set at 1.88 log_10_ GenEq/g. Samples with counts under the threshold were set at 0.94 log_10_ GenEq/g. No significant differences were observed between groups.

In the second experiment, the YP9817 antigen, which reduced *Campylobacter* loads the most in trial 1, was also tested using DNA vaccination alone on day 5 or protein vaccination alone on day 12. As shown in Fig 3A, vaccinated groups did not develop a specific humoral response since IgY levels were similar in both vaccinated and respective control groups. A slight increase in IgY levels was observed in all the groups from day 21 to day 41 due to *Campylobacter* infection on day 19. These results indicate the importance of the DNA prime/protein boost protocol in inducing a specific humoral response (Fig 1B), since DNA or protein alone did not induce a specific immune response (Fig 3A). Also, no decrease in *Campylobacter* loads was observed in the vaccinated groups (Fig 3B); birds were colonized at 7.04 log_10_ CFU/g for the DNA-YP9817 group and 7.87 log_10_ CFU/g for protein-YP9817 group, levels similar to those of the control groups. However, in the second trial, even after DNA prime/protein boost vaccination the YP9718 antigen did not protect birds against *Campylobacter* (Fig 2B). These last data notwithstanding demonstrated that DNA prime and protein boost vaccinations are both needed for the induction of a specific immune response.

## Discussion

The vaccination of broiler chickens against *Campylobacter* is one of the strategies identified to decrease avian intestinal loads and, consequently, to reduce the incidence of human campylobacteriosis. Many vaccine studies have been undertaken in the past few decades in order to reduce *Campylobacter* loads in chickens, but there is as yet no commercial avian vaccine against *Campylobacter*. The study described in this paper assessed the immune and protective power of newly identified antigens by the reverse vaccinology strategy [25]. Of the 14 antigens identified, six—YP437, YP562, YP1115, YP9769, YP9817, and YP9838—were produced for DNA and protein vaccines. Two other vaccine candidates were first selected for *in vivo* testing (YP_001000153.1 and YP_001000945.1), but their production as a protein was not successful. After several trials, these antigens appeared to harm the *E*. *coli* XL1-Blue bacteria in which they were produced. After initiating protein production, bacteria were totally lyzed at the pellet collection step. The plasmid sequences were checked to ensure they were correct. Cloning in pQE-TS failed for another three antigens (YP_001000383.1, YP_001000935.1, and YP_001001008.1). Since antigens are *Campylobacter* proteins, the authors chose the bacterial system for protein production to conserve the limited post-translational modifications. However, other strategies could be adopted for the production of vaccine candidates using baculovirus or eukaryote expression systems, or using other expression plasmids or bacteria strains.

The vaccine regimen used to evaluate the protective potentials of the selected antigens consisted of a DNA vaccination on 5-days old chicks followed by protein vaccination at the age of 12 days. An oral *Campylobacter* challenge was peroformed on 19-days old chicks. The DNA prime/protein boost vaccine regimen is essential since vaccinations with DNA or protein alone did not induce a specific humoral response. According to this protocol, the tested antigens induced an anti-*Campylobacter* response in serum that the DNA/protein control group did not. Only the YP9769 antigen did not show statistically significant IgY levels in serum and seemed to be non-immunogenic. However, it is important to note that the ELISA used in this study was developed to screen the immune potential of several vaccine candidates in comparison to the DNA/protein control group. The plates were coated with an extract of total *Campylobacter* proteins and there is no evidence that all the proteins were present at high enough levels to be detected by the specific antibodies. This could explain why no antibodies against the YP9769 protein were detected. Individual ELISAs should be developed and optimized to detect immune responses against individual antigens if the test against all the proteins does not detect antibodies.

In this study, YP437, YP562, YP9817, and YP9838 antigens appeared to be potential vaccine candidates since their administration to chicks led to a significant decrease in *Campylobacter* load, up to 4.2 log_10_ CFU/g. These results are very promising because such reductions are equal or higher than those estimated to reduce the risk of human campylobacteriosis by 76 to 100% [6, 7]. However, these results were difficult to reproduce. In a second trial, cecal load reductions results were not statistically confirmed despite a strong immune response. Thus, additional work is needed to understand and improve the vaccine efficiency. Another study have demonstrated the strong protective effect of a glycoconjugated vaccine [19] against *Campylobacter* colonization in SPF Leghorn chickens. Like Layton et al. (2011) using Omp18/CjaA antigens vectored by *Salmonella*, we observed, even if this was not statistically confirmed in a second experiment, significant reductions of *Campylobacter* loads in broilers, which are the main birds involved in campylobacteriosis.

The protective potential of YP9817 and YP9838 observed in the first experimental trial was not confirmed in the second one despite similar specific immune responses for both *in vivo* experiments. One hypothesis to explain this difference is that YP9817 and YP9838 protein vaccine production batches were not the same for the two experimental studies and it cannot be excluded that protein conformational modifications for example did not occur between both batches; especially as in the case of YP437 and YP562 antigens, the same protein productions were used in both *in vivo* trials and quite more similar results were obtained. It also could be due to the inter-individual variability observed in the second trial. Indeed, high inter-individual variability is a recurrent problem of *in vivo* studies. Similar results [26, 30, 31] have been mostly associated with intrinsic factors related to broilers rather than to environmental factors related to animal husbandry, since temperature, light or ventilation parameters were all controlled [31]. Despite this variability, YP437 and YP562 groups showed a non-significant decrease in *Campylobacter* loads.

When antigens were evaluated individually, chicks received 300 μg of plasmid DNA and 100 μg of recombinant proteins. The combination of antigens performed by injecting 50 μg of each DNA plasmid and 16.7 μg of each recombinant protein induced no decrease in *Campylobacter* numbers even if the production of IgY antibodies was as high as the ones obtained for individual antigens that permitted to reduce significantly cecal *Campylobacter* colonization. This absence of protective effect of the pool of antigens could be explained by the fact that the induced immune responses targeted different *Campylobacter* antigens and the amount of each antigen-targeted immune responses were not sufficient to neutralize bacteria. Furthermore, the production of IgY against *Campylobacter* in response to vaccination and challenge was of the same magnitude for 5 of the 6 antigens evaluated in first experiment, as well as for the pool of antigens. Nevertheless the levels of protection induced by these antigens were not the same. It varied from no impact on the *Campylobacter* cecal load to significant decreases of up to 4.2 log_10_ CFU/g of cecal content. Taking all these results together, this means that additional mechanisms to antibodies may be involved in protection against *Campylobacter*, as it was recently suggested [32].

Among the tested antigens, three were described as flagellar antigens with extracellular localizations. YP562 (FlgL), YP1115 (FlgK), and YP9769 (FlgE-1) have already been described as virulence factors involved in *Campylobacter* motility [33] and characterized *in vitro* [34]. Both FlgK and FlgE-1 proteins induced a widespread reaction in chicken sera (more than 70% of tested sera), whereas the FlgL protein immune-stained with only 21% of the tested sera. In our study, the three antigens showed lower *Campylobacter* loads in vaccinated birds but the differences were not significant except for the FlgL antigen in one experiment.

The other three antigens tested (YP437, YP9817, and YP9838) are not as yet fully described. According to bioinformatics predictions [25], they are localized on the outer membrane protein of *Campylobacter*, but their role and involvement in *Campylobacter* virulence have not as yet been investigated. Preliminary *in silico* analyses have shown that YP437 and YP9838 could be involved in the secretion and activation of hemolysin and protein-protein interactions respectively. Thus, YP437 could act as a virulence factor since *Campylobacter* hemolysin could contribute to its pathogenicity [35, 36]. *In vitro* and *in vivo* characterization is needed to confirm these predictions.

This study highlights the advantages of reverse vaccinology [37], a strategy used here to identify vaccine candidates to be tested [25], as among the six individually-tested vaccine candidates during the first trial, five induced an immune response in chickens and four reduced *Campylobacter* loads compared to the DNA/protein control group. However, despite the reproducible induced immune response obtained for the tested antigens during the second trial *Campylobacter* reductions were not reproduced for 2 antigens or not statistically confirmed for two others due to high inter-individual variations in *Campylobacter* loads. This recent strategy has already proven its efficiency, firstly with the development of a vaccine against B serogroup *Neisseria meningitidis* [38, 39]. Many studies have since been successfully conducted [40–43]. In this study, we have demonstrated the immune and protective powers against *Campylobacter* of four new antigens (YP437, YP562, YP9817, and YP9838) even if this was not statistically confirmed in a second experiment. The reverse vaccinology strategy enabled us to quickly identify and assess these antigens. However, these new and promising results need to be further investigated in confirmation and refinement studies in order to develop an avian vaccine with potential for tackling *Campylobacter* in the broiler production chain.

## Supporting information

S1 Table Ethic comments. *Plos One* Humane Endpoints Checklist.
(DOCX)
