# Supplementary material for: Promising new vaccine candidates against Campylobacter in broilers
Source: PLoS One. 2017 Nov 27;12(11):e0188472. doi: 10.1371/journal.pone.0188472 (PMC5703506; doi:10.1371/journal.pone.0188472)
Supplement: S1 Table — Plos One Humane Endpoints Checklist. (DOCX) [file pone.0188472.s001.docx]

*PLOS ONE* Humane Endpoints Checklist

*PLOS ONE* manuscript number: _______________________

**Complete the following if your study design includes death of a regulated animal as a likely outcome or planned experimental endpoint. Please also include all information in the Methods section of your manuscript.**

**ITEM 1.** **Describe whether humane endpoints* were used for all animals involved in the study.**

|  | **Recommendation** | **Section/Paragraph** |
| --- | --- | --- |
| **If humane endpoints* were used, report the following:** | | |
| **1** | **The specific criteria used to determine when animals should be euthanized** | **Not Applicable. Campylobacter is not pathogenic for the chickens** |
| **2** | **Once animals reached endpoint criteria, the amount of time elapsed before euthanasia** | **Not Applicable. Campylobacter is not pathogenic for the chickens** |
| **3** | **Whether any animals died before meeting criteria for euthanasia** | **Not Applicable. Campylobacter is not pathogenic for the chickens** |
| **If humane endpoints* were not used, report the following:** | | |
| **1** | **A scientific and ethical justification for the study design, including the reasons why humane endpoints could not be used, and discussion of alternatives that were considered but could not be used** | **See line 136**  **Animal experiments were performed using protocols approved by** the ANSES ethical committee (ethical protocol no. 14-065, agreement no. 09/12/14-1B).  **Campylobacter is not pathogenic for the chickens** |
| **2** | **Whether the institutional animal ethics committee specifically reviewed and approved the anticipated mortality in the study design** | **Not Applicable. Campylobacter is not pathogenic for the chickens** |

**ITEM 2.** **Include the following details of the study design and outcomes.**

|  | **Recommendation** | **Section/Paragraph** |
| --- | --- | --- |
| **1** | **The duration of the experiment** | **see starting Line 156:**  **42 days (+/- 1 day)** |
| **2** | **The numbers of animals used, euthanized, and found dead (if any); the cause of death for all animals** | **see starting line 143:**  **314 chickens in total, 2 experiments. 2 to 4 birds / placebo group euthanized on day 22 to control colonization. The rest euthanized on day 42 for Campylobacter counts on caeca.**  **20 out of 314 chickens were found dead few days after hatching (corresponding to the normal residual mortality in avian livestock) or euthanized because of rickets or malformations.** |
| **3** | **How frequently animal health and behavior were monitored** | **see starting line 138:**  **Observed daily during the first week of life and every working days after. Body weights and feed consumption monitored by the Avian Breeding and Experiment Department** |
| **4** | **All animal welfare considerations taken, including efforts to minimize suffering and distress, use of analgesics or anaesthetics, or special housing conditions** | **Yes. This is indicated in the experimental protocol submitted to the ethical committee** |
| **5** | **Any special training in animal care or handling provided for research staff** | **The whole staff taking care of or handling animals was trained.** |

***Definition of a humane endpoint**

A humane endpoint is an experimental endpoint at which animals are euthanized when they display early markers associated with death or poor prognosis of quality of life, or specific signs of severe suffering or distress. Humane endpoints are used as an alternative to allowing such conditions to continue or progress to death following the experimental intervention (“death as an endpoint”), or only euthanizing animals at the end of an experiment. Before a study begins, researchers define the practical observations or measurements that will be used during the study to recognize a humane endpoint, based on anticipated clinical, physiological, and behavioral signs. These may include, for instance, body temperature or weight changes, tumor size or appearance, abnormal behaviors, pathological changes, ruffled fur, reduced mobility, body posture, or expression of specific body fluid markers. Please see the NC3Rs guidelines for more information.

**ARRIVE Guidelines**

*PLOS ONE* encourages authors to follow the [Animal Research: Reporting of In Vivo Experiments (ARRIVE) guidelines](http://www.nc3rs.org.uk/arrive-guidelines) for all submissions describing laboratory-based animal research and to upload a completed [ARRIVE Guidelines Checklist](http://www.nc3rs.org.uk/sites/default/files/documents/Guidelines/NC3Rs%20ARRIVE%20Guidelines%20Checklist%20%28fillable%29.pdf) to be published as supporting information.
